# Non-Enzymatic Decomposition of Collagen Fibers by a Biglycan Antibody and a Plausible Mechanism for Rheumatoid Arthritis

**DOI:** 10.1371/journal.pone.0032241

**Published:** 2012-03-13

**Authors:** Olga Antipova, Joseph P. R. O. Orgel

**Affiliations:** Pritzker Institute of Biomedical Science and Engineering, Illinois Institute of Technology, Chicago, Illinois, United States of America; Massachusetts Institute of Technology, United States of America

## Abstract

Rheumatoid arthritis (RA) is a systemic autoimmune inflammatory and destructive joint disorder that affects tens of millions of people worldwide. Normal healthy joints maintain a balance between the synthesis of extracellular matrix (ECM) molecules and the proteolytic degradation of damaged ones. In the case of RA, this balance is shifted toward matrix destruction due to increased production of cleavage enzymes and the presence of (autoimmune) immunoglobulins resulting from an inflammation induced immune response. Herein we demonstrate that a polyclonal antibody against the proteoglycan biglycan (BG) causes tissue destruction that may be analogous to that of RA affected tissues. The effect of the antibody is more potent than harsh chemical and/or enzymatic treatments designed to mimic arthritis-like fibril de-polymerization. In RA cases, the immune response to inflammation causes synovial fibroblasts, monocytes and macrophages to produce cytokines and secrete matrix remodeling enzymes, whereas B cells are stimulated to produce immunoglobulins. The specific antigen that causes the RA immune response has not yet been identified, although possible candidates have been proposed, including collagen types I and II, and proteoglycans (PG's) such as biglycan. We speculate that the initiation of RA associated tissue destruction *in vivo* may involve a similar non-enzymatic decomposition of collagen fibrils via the immunoglobulins themselves that we observe here *ex vivo*.

## Introduction

The major parts of the joint which are affected most prominently in RA, are articular cartilage and the synovium. Collagen type II fibrils are major structural elements of the cartilage ECM and the cartilage-like notochord of cartilaginous fishes. They form 67 nm periodic fibrils and fibers, with the participation of proteoglycans (PG's), which bind collagen by their core protein and regulate collagen fiber diameter through their anionic glycosaminoglycan (AGAG) chains. These collagen-PG interactions are also essential for fiber/fibril stability and determine a number of their mechanical properties [1]–[5]. In cartilage, collagen type II also aggregates with other collagen types (I, V, IX and XI) [6] and PG's to form complex fibrillar meshworks, in contrast to the simple arrangement in notochord. Although the type II collagen fibrils themselves are indistinguishable between the two tissues [7]–[9]. Aggrecan in complex with hyaluronan is embedded within these meshworks and accumulates a substantial amount of water (70% of cartilage mass) by their highly negative charge.

The synovium, or synovial membrane, is a thin sheet of vascularized mesenchymal tissue that surrounds the joint cavity and produces synovial fluid, which is responsible for joint lubrication and chondrocyte nutrition, since avascular cartilage is impermeable to oxygen and nutrients in mature joints [10]. The ECM of synovium is composed of collagen fibrils (types I, III, and V, type II is not present) of relatively small diameter (30 nm) with 67 nm periodicity and thin filaments (10 nm) of collagen type VI (with 100 nm periodicity), which are integrated with hyaluronan, fibronectin, and fibrillin, providing tissue permeability and structural integrity [10].

PG's, such as the small leucine rich repeat proteins (sLRRP's) decorin and biglycan, are essential for stabilization of fibril-bundle structures [11] and for conveying compression resistance together with hyaluronan and aggrecan. Here we report that biglycan bound type II collagen fibril-bundles (or fibers, also known as thick fibrils of 30–50 nm diameter) are decomposed into discrete fibrils (also known as thin-fibrils of ∼10–15 nm diameter) through the action of anti-biglycan antibody, even in the absence of cells, enzymes, other antibodies and in the presence of enzyme inhibitors. This antibody induced process results in the breakdown of notochord and the appearance of thin-fibrils in cartilage samples.

## Results and Discussion

### Extracellular matrix degradation during RA

Part of the RA inflammatory response is release of proteases, such as matrix metalloproteinases (MMPs) and “a disintegrin and metalloproteinases with thrombospondin motifs” (ADAMTSs), which digest the ECM of the synovium and cartilage. The major targets in cartilage are collagen type II, PG's (decorin, biglycan) and aggrecan; their remnants were detected in body fluids of RA patients [12]–[15]. As a result of Polgar proteolysis, the superficial layer of cartilage is destroyed [16] and its structure and biomechanical properties are altered. The loss of PGs and aggrecan leads to a decrease of water molecules in cartilage and therefore resistance to compression, but it may also affect the stability of collagen fibrils and makes them more vulnerable to MMPs.

**Figure 1:**
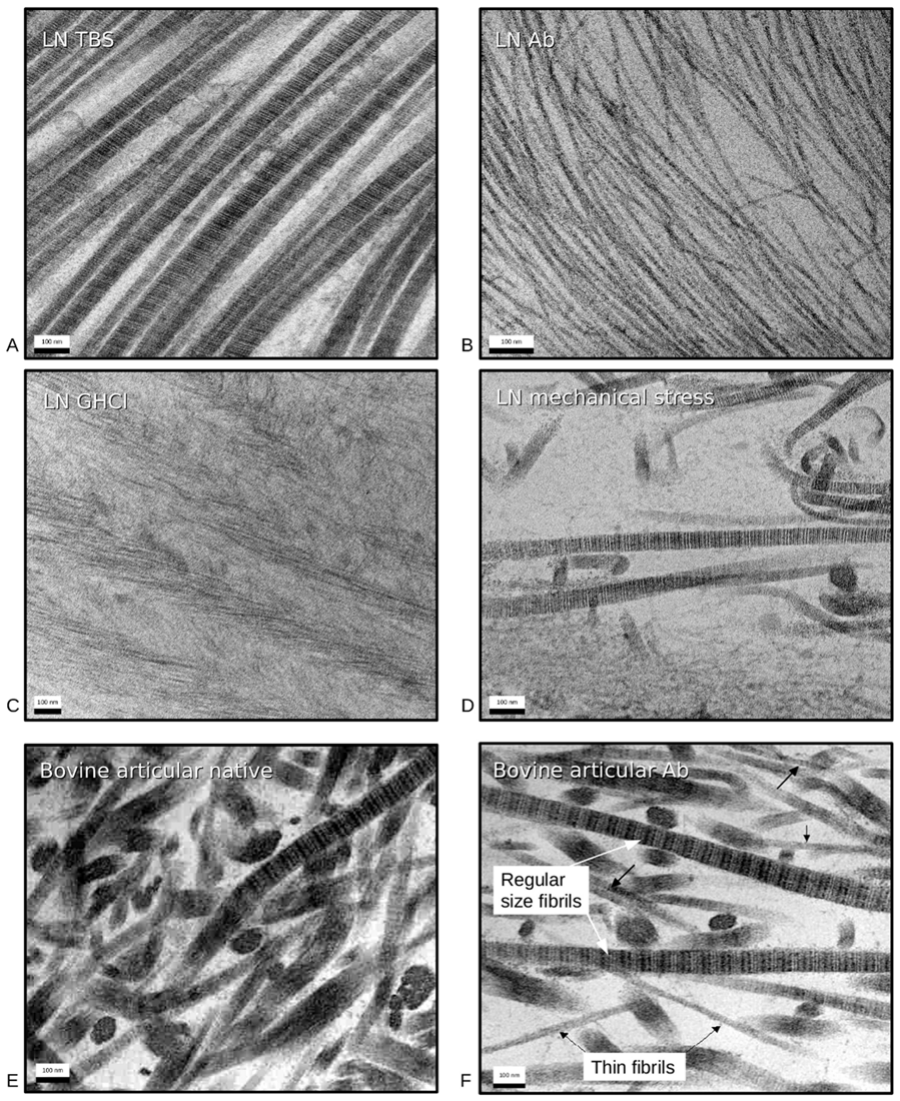
Decomposition of collagen type II fibrils in lamprey notochord viewed with TEM (A–D) and bovine articular cartilage (E and F). A) Native (prior to fixing) type II collagen fibrils, incubated in TBS as control for fibril samples shown in B–D. Average fibril size is around 35 nm. B) Collagen type II fibrils following short incubation with anti-biglycan antibody. Fibril diameter is 10–15 nm. C) Collagen type II fibrils following incubation in GHCl. Although severely disrupted, the fibril decomposition appears less complete than that of the antibody incubation (B). D) Collagen type II sample following mechanical disruption. Disruption of native fibril structure is highly localized, with large sections still intact. E) Native bovine articular cartilage (prior to fixing and staining for TEM). F) Bovine articular cartilage post 1 hour treatment with anti-biglycan. Black arrows point to thin-fibrils, white arrows point to normal sized thick-fibrils.

**Figure 2:**
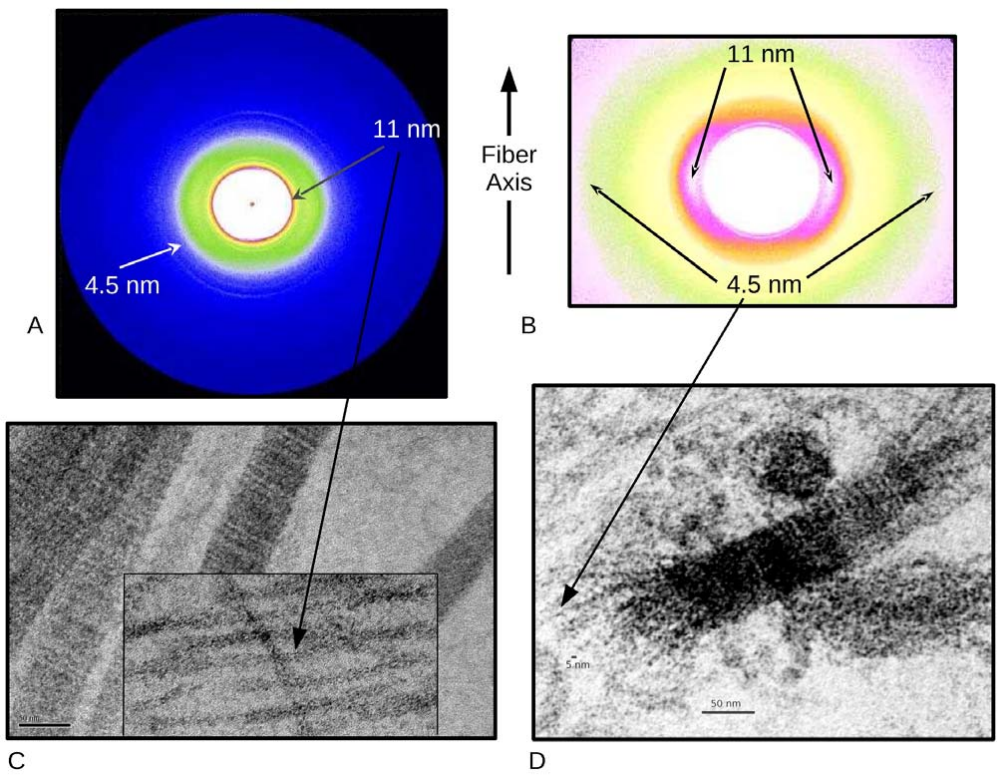
Type II collagen fibrils decomposed into their basic aggregates (viewed via X-ray diffraction and TEM). Some parts of the antibody treated samples maintain a loose alignment of the thin-fibrils allowing them to be analyzed with small angle X-ray diffraction (A), and insert B. An 11 and 4.5 nm packing function are apparent, which appear to correspond to the approximate diameter of the thin-fibrils (insert of C) and microfibrils (D). Native thick fibrils are shown in C as a comparison to the decomposition product (thin-fibrils).

**Figure 3:**
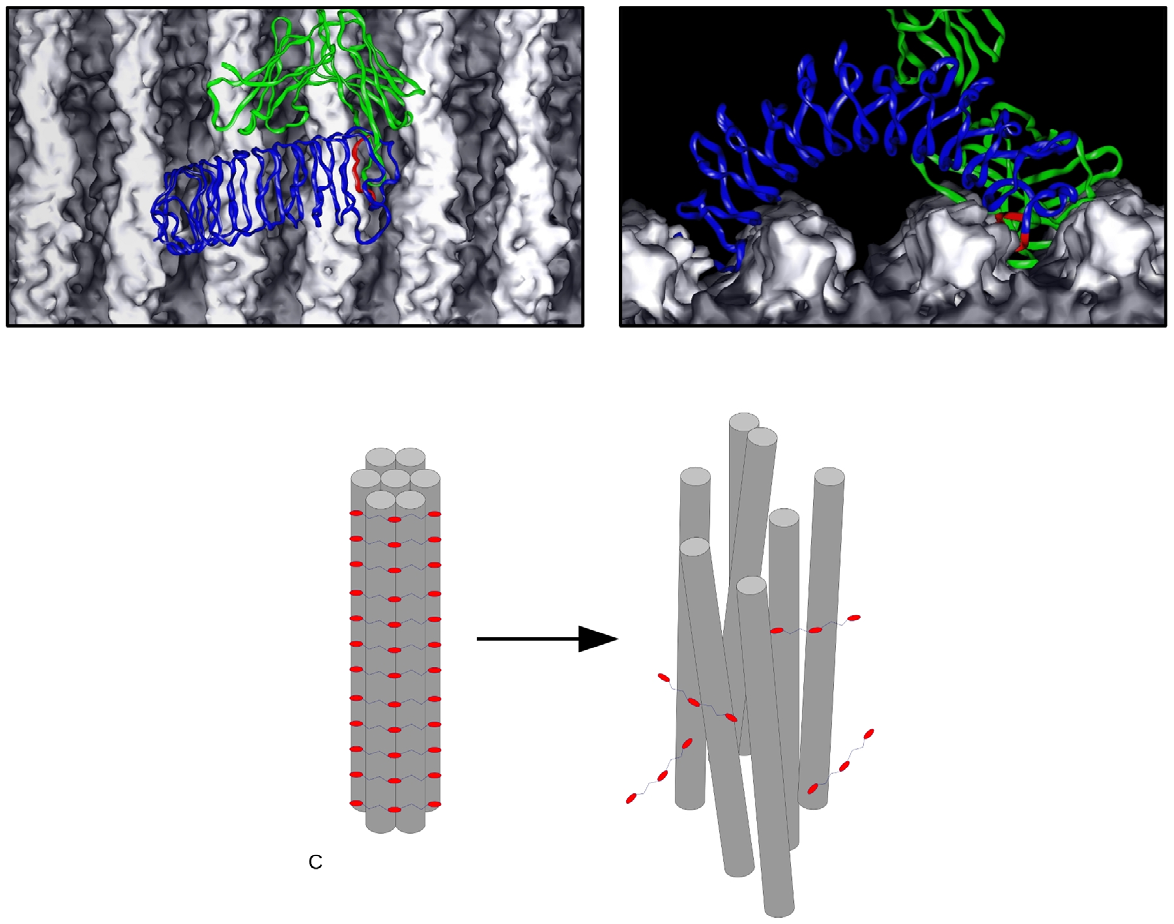
Model of antibody action on type II collagen fibrils. Coordinate models of the biglycan-type II collagen fibril complex based on the decoron-type I collagen fibril structures published recently (1) are shown with a model Fab (green) unit attaching to the biglycan (blue) epitope (colored red, A and ‘top’ view B). Because the epitope is located within a solvent filled channel of the collagen fibril [33], there is room for loops of the fab to dock with it, but its close proximately to the fibril-PG hydrogen bonding network located between the collagen fibril surface and the concave side of the PG-core proteins structure (1) may disrupt the positive interactions and dislodge the core protein from the fibril. Leading to the debundling of thick-fibrils into their constitutive thin-fibrils (C).

**Figure 4:**
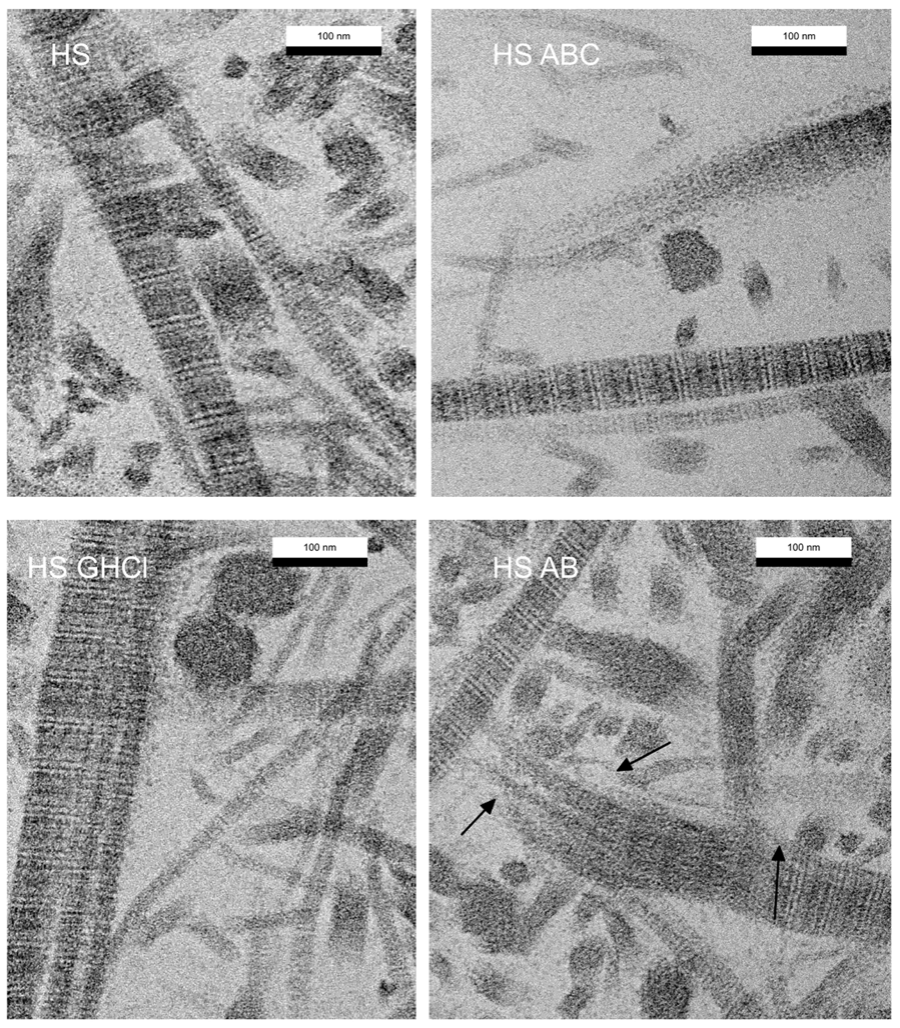
TEM images of human articular cartilage preparations. A) Section of native human articular cartilage, incubated in TBS, that has collagen type II fibrils of regular 30–50 nm diameter (control for samples B–D). B) Section of human articular cartilage treated with ABC lyase for 24 h with some thin fibrils of collagen type II. C) Section of human articular cartilage treated with Guanidine hydrochloride for 24 h with presence of thin 10–15 nm fibrils and normal thick fibrils (fibril bundles). D) Section of human articular cartilage, treated with anti-biglycan antibody for 24 h, shows some collagen type II thin fibrils as well as fibrils of regular 30–50 nm diameter. Arrows point to decomposing fibrils.

**Figure 5:**
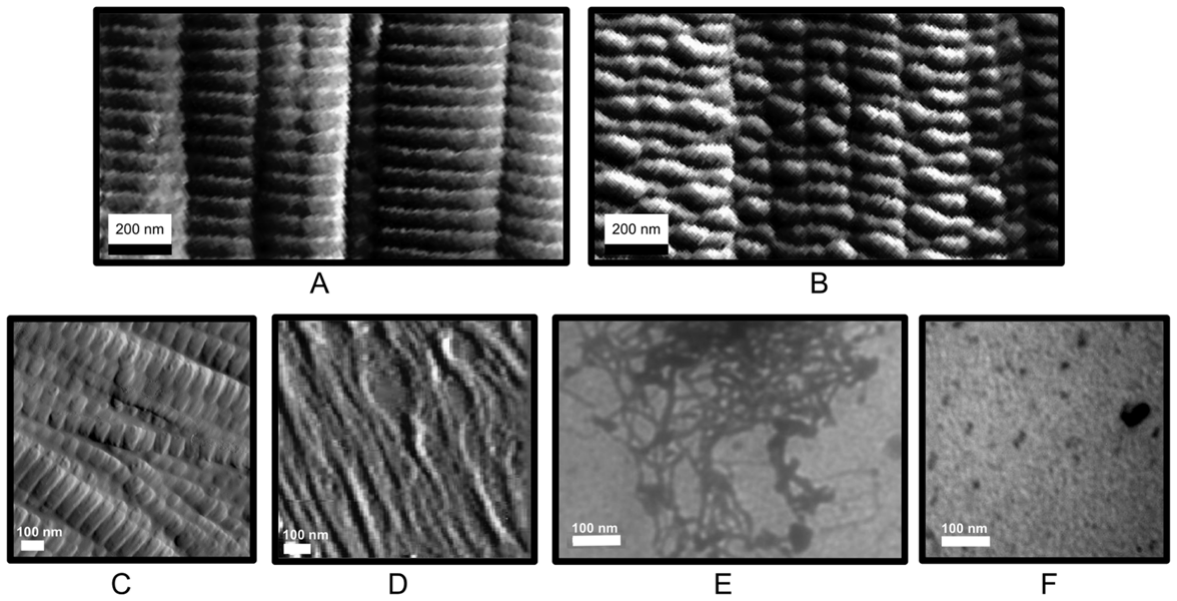
AFM and TEM images native and Ab treated rat tail tendon and lamprey notochord treated samples. A) Control AFM data of type I collagen fibrils: native type I fibrils. B) Control AFM data of type I collagen fibrils: Anti- PG core protein antibody conjugated with 30 nm particles attached to type I fibrils. Note that the fibrils are intact and that the gold particles are clearly discernible as densely packed globules. C) AFM image of native lamprey type II fibrils. D) AFM image of lamprey type II fibrils after treatment with gold particle conjugated anti- biglycan antibodies as in B. Compare with B. No gold particles are visible, whilst in the type I collagen control, they are clearly imaged. E) Unstained TEM of lamprey derived collagen fibrils following anti-biglycan treatment. The antibodies in this preparation were conjugated to 10 nm gold particles and were still able to decompose the thick fibrils into thin-fibrils, but no gold particles are visible after the preparation is washed in TBS. F) Unstained TEM of biglycan aggregates attached to gold particle conjugated antibodies. Gold particles are clearly visible as dense black spots that do not appear in E.

Initial ECM degradation, however, may occur in the absence of proteases. Severe mechanical loads as well as changes in pH may cause cartilage fibrillation [17], [18]. Depleted PG content is observed in the articular cartilage of RA patients accompanied with fibrillar fragmentation [19]. Whilst elevated levels of biglycan antibodies have been detected in serum and synovial fluid of RA patients [20] and they are considered to be early markers of this disease. However the exact role of these antibodies in initiation and development of the drastic changes RA causes to cartilage has remained unclear as does the specific mechanism of tissue destruction. Here we present evidence of the potent effect of an antibody to biglycan on cartilage-like (lamprey notochord) and articular cartilage tissues at physiological pH and in the absence of cell associated or free enzymes. In the presence of this antibody, type II collagen thick-fibrils are quickly decomposed into much smaller species inducing irreversible damage to the tissues, as visualized by electron microscopy, atomic force microscopy and X-ray diffraction (FIGS. 1, 2, 3, 4, and 5). Apart from the possible significance of this observation to RA, we are unaware of any previous reports of autoimmune associated antibodies being directly responsible for inducing such destruction of vertebrate and mammalian connective tissues.

### Antibody induced tissue decomposition

Native fibrils of lamprey notochord do not show any detectable difference between its collagen type II fibers and those seen from the tissues of mammals [21] (FIG. 1). They have the same diameter of about 35 nm and typical positive staining pattern, although there are some differences in cellular and proteoglycan content, as well as tissue architecture. Lamprey notochord appears to have a very specific cell distribution (layered, outside of the bands of extracelluar material), whereas cartilage cells are embedded in a collagen meshwork and can be seen throughout the whole tissue. The PG content of lamprey notochord is fairly simple containing only biglycan type I and II (which are very close in sequence to the bovine or human biglycans, and hence structurally related to decorin and fibromodulin), in contrast to the mammalian cartilage which has several types of PG's and glycoproteins present [22], [23].

TEM images of lamprey tissues treated with the biglycan antibodies showed (FIGS. 1–2) a striking difference from the native fibers: 1) much thinner collagen fibers about 11 nm in diameter are present through-out the samples or more accurately, there was a notable absence of native thick-fibrils (Tables S1 and S2) and 2) the ECM became significantly less well ordered. The antibody treated notochord (FIG. 1B) after treatment was very delicate and easy to deform, unable to withstand even minimal strain (<5%) without its destruction. Mammalian tissues were not affected so dramatically (FIGS. 1 and 4), but they also showed the appearance of thin fibrils and disrupted fibers in the presence of the antibody, not present in the control (pre-treated) sections in such quantities (FIG. 1). The difference in the relative effect on the two animal tissues (cartilage vs. notochord) is presumably due to the mammalian cartilage possessing a wider repertoire of sLRRP's that are either completely or partially resistant to the effects of the anti-biglycan antibody (see below). Lamprey notochord with only biglycan is therefore more susceptible to any putative effects of its antibody. Regardless of the difference in scale of effect, the same unexpected observation was made between the tissues: Significant and rapid non-enzymatic decomposition of collagen fibrils occurs in the presence of the anti-biglycan antibody. Large BG core protein sized structures observed in TEM of incubating solution with presence of gold particles. Biochemical analysis shows significantly elevated levels of BG in this solution relative to non-ab containing controls (see SI methods S1, Tables S3 and S4, and FIG. 5).

We suggest, that anti-biglycan antibodies attach to biglycan core-proteins on the surface of thick-fibrils and that this interaction disrupts the bonding between the core-protein and the collagen molecules comprising the fibrils. Even though GAG bridges remain intact, the loosening of the biglycan core-protein – collagen interaction ‘unties the string’ that holds the thick-fibrils together (FIG. 3). This further implies that ‘thick-fibrils’ are in fact bundles of thin fibrils (i.e. the ‘fibril bundles’ discussed throughout this study). Whereas a ‘thin-fibril’ [24] is an irreducible collagen fibril (without enzymatic digestion or mechanical force) formed from closely packed collagen molecules and held together through collagen-collagen interactions such as lysine-hydroxylysine bonds.

### Controls

In order to test the hypothesis that disruption of the biglycan core-protein – collagen interaction accounts for the fibril-debundling, lamprey notochord samples were treated with Guanidine hydrochloride and ABC lyase respectively as a positive control. Guanidine hydrochloride causes protein denaturation, therefore its action would mimic the hypothesized action of the biglycan antibody, albeit in a more caustic and less specific manner. TEM images of Guanidine treated notochord and cartilage illustrated the same type of degradation of collagen fibrils, although the incubation time had to be much longer (24 h instead of 1 h for the antibody) to achieve a similar degree of decomposition. In contrast, ABC lyase removes the AGAG chain from the protein core and should produce the similar results albeit via a different structural mechanism. Thin fibrils in both notochord and articular cartilage samples were seen in TEM images after this treatment, although damage was relatively mild in comparison to the antibody-mediated decomposition. In addition to these chemical methods of tissue degradation, mechanical degradation was also examined. Friction was applied to native lamprey notochord tissues and the results of this damage were analyzed by TEM and compared with the other experiments. Mechanical impact, used for this study, may correspond to damage of the articular cartilage due to trauma or normal wear. The presence of thin fibrils were observed (absence of regular 30–50 nm fibrils) in certain areas, which had higher load, although some parts of tissue still had normal architecture in comparison to undamaged, non-treated control samples. Finally, significant tissue degradation (and biglycan release) was observed in the presence of protease inhibitors but not in the presence of alternative antibodies such as anti-collagen (see SI methods S1).

### Notochord structure: differences in scale of the effect of anti-biglycan on lamprey vs. mammalian tissues

Lamprey notochord is a cartilage-like tissue that spans the length of the chordate back, located beneath and parallel to the central nervous system between the brain and tail. Although it is the main axial skeleton at the embryonic stage, the notochord is replaced by the vertebral column in most vertebrates. However, in some chordates it remains into adulthood (e.g., lamprey, lungfish, sturgeon, and some sharks). The mature notochord contains a soft cellular inner part, surrounded by protective fibrous sheath, composed of three layers: inner basal lamina, thick collagenous (cartilage-like) layer, and elastic filamentous membrane [25]. The composition of collagenous part of lamprey notochord is rather simple compared to articular cartilage, which makes it very convenient to study. It has two main collagen type II fiber orientations: circular (perpendicular to the main body axis) and longitudinal (parallel to the main axis of the body). Longitudinally organized fibers are located at the outer layer and are the most prevalent. The fibrillar architecture is supported by the lamprey biglycan. Although the organization of articular cartilage and lamprey notochord seem to be different, the structure of collagen type II fibrils in both of them is indistinguishable [25] and their sequence is highly homologous.

Variations in the different level of damage (thin/normal collagen type II fibrils), obvious in TEM images of mammalian and lamprey tissues, can be explained by differences in the molecular composition of these tissues that in turn influences the tissue architecture. Lamprey notochord contains primarily collagen type II and biglycan. Human and bovine articular cartilage contain biglycan, fibromodulin, decorin, and other ECM molecules, which regulate fibrillogenesis, fiber diameter, support fibers, and give the tissue specific mechanical properties. Cartilage and meniscus contain more biglycan than decorin and the ratio changes from zone to zone. The superficial zone contains about 32% of decorin and 38% of biglycan of all PG content, the inner deep zone contains about 23% of decorin and 53% of biglycan of all PGs, and middle zone has 28% of decorin and 52% of biglycan of all PG's [26]. As a result these tissues can be expected to be more resistant to short duration (1–2 hours) antibody treatment. Due to the presence of decorin, which seems to be stable in the presence of the antibody (this antibody has higher affinity to biglycan, than decorin). But longer exposure of cartilage tissues to biglycan antibodies has a stronger effect on cartilage collagen fiber disassociation, presumably because the antibodies need more time to penetrate to deeper layers where biglycan is predominant.

### Antibody concentration and effect

Although our observations are *ex vivo*, it is worth noting that human autoimmune disease antibody concentration levels have been reported in body fluids at levels similar to or higher than we have applied in our experiments [27]–[29], although we also observed this effect in lower starting concentrations (see SI methods S1). What is more, the observed fibril decomposition permeated whole tissue samples immersed in the antibody containing vials. The concentration of the antibody in the tissue interior must have been substantially lower than in the surrounding solution, and yet, still caused tissue degradation. That the effect was observed in so short a period of time (in notochord) is also significant.

Given that the concentration of the antibody used may be physiologically relevant, the nature of its disruptive affect may be the same *in vivo* as our *ex vivo* experiments, be that through competitive bond disruption or steric repulsion or both. However, as FIG. 3 indicates, it is quite unlikely that more than one antibody molecule may locate to one biglycan core proteins epitope, making the steric repulsion consideration less likely. Especially in light of published studies that indicate sLRRP's conformations are not easily given to significant changes to their arching structure but do seem to be sensitive to the environments effect on their inner-core to collagen fibril hydrogen bonding network [1], [4]. The fact that we observed some sensitivity of tissues to certain salts in buffer systems (see SI methods S1and PBS) and that simple denaturing conditions (see guanidine hydrochloride GHCL results) were less effective than the application of the antibody may be considered collaborating evidence in favor of the interaction interference effect of the anti-biglycan antibody. It has long been known that pH causes collagen fiber bundles to disassociate into smaller species [30], [31], the latter reference speculating that the sLRRP proteoglycans are sensitive to both salt and pH conditions. That we observed this affect at natural pH in a phosphate based buffer system would seem to collaborate these prior considerations. That we did not observe fibril-bundle degradation in TBS controls and poorer destruction of fibril bundles in TBS via GHCL or enzyme catalysis, suggests a novel effect of the anti-biglycan antibody against type II collagen fiber bundles. That the thin-fibrils were not further degraded but remained indefinitely stable rules out the possibility of a general or collagen specific proteinase. The significantly elevated levels of biglycan detected in the anti-biglycan solution in which the tissue were incubated relative to controls without the anti-antibody, supports the hypothesis that anti-biglycan causes the disassociation of biglycan from thick-fibrils.

### Collagen fibril MMP cleavage site and collagenase-interaction domain

Collagen fibrils are assembled in such way that the MMP collagenase cleavage-site is protected by the C-telopeptide in folded conformation [32]–[34]. This folded C-telopeptide corresponds to the X3 ridge in type I collagen, and appears to have an analogous structure in type II collagen as observed from AFM and X-ray diffraction data [32], [33]. The C-telopeptide is usually cross-linked covalently with neighboring collagen molecules, that helps makes its conformation very stable. In order to gain access to the MMP cleavage-site, other proteases have to cleave the C-telopeptide first. This process is very slow with MMP1 alone due to a very low number of available cleavage sites (presumably at the fibril tips were collagen packing is looser). Our results show that the biglycan antibody can induce the process of collagen fiber decomposition in cartilage-like tissues. Even a short, one-hour exposure of lamprey notochord to biglycan antibody completely altered the structure of the collagen matrix. In fact there were only few areas in the treated notochord that were not altered dramatically (fibrils disassociated, but stayed close and parallel to each other) and therefore were capable of yielding diffraction patterns (FIG. 2). Even these ‘less affected’ tissue area's showed significant structural changes and most importantly, almost uniform fibrillar decomposition. Our preliminarily data (not shown) of enzymatic digestion of antibody incubated notochord showed that the tissue was decomposed at least a factor of 2 times faster than the controls (at 4°C, compared with MMP1, trypsin and pepsin), indicating that previously exposed cleavage sites were becoming exposed due to the action of the antibody on BG (i.e because fibril-bundles are being disrupted, yielding their constituent thin-fibrils and biglycan, see FIGS. 3 and 5).

### A speculative molecular based mechanism for RA associated tissue destruction

The observed tissue destruction in lamprey notochord and analogous fibrillar decomposition in articular cartilage as a response to anti-biglycan treatment, may have significance to human health and aging. Elevated levels of anti-biglycan antibodies are detected in the body fluids of arthritis patients [20], and are considered early markers of this disease, but their specific role has not previously been clarified. The presence of thinner fibers and high concentration of collagen cleavage products are also connected to RA events [35]. Our results from biglycan antibody treatment – cartilage/notochord collagen interactions visualized by TEM and supported by X-ray diffraction based evidence of collagen fibril architecture and collagenolysis [32], [33], [36], [37] can be used to propose a mechanism of collagen matrix degradation by MMPs, initiated by autoimmunity. It is known that the interaction between collagen and cartilage PG's strongly depends on their specific conformation [38]. Thus, even small changes may be enough to disrupt the connection between biglycan and collagen molecules. Recent work to define the nature of the interaction between the biglycan homologue, decorin, and the type I collagen fibril suggests how this might occur; a disruption to the hydrogen bonding network of the PG-core protein-collagen fibril interface [4] in the region of the anti-biglycan antibody epitope (see methods). Therefore we speculate that the process of RA tissue destruction could begin here or be one of the early factors leading to the pathological cascade of RA:

1. Antibodies against biglycan (and perhaps other sLRRP protein cores, although biglycan appears to be the most vulnerable as decorin appears unaffected despite its close homology) binds to the PG-core protein and disrupts the hydrogen-bonding network on the interior of the concave surface that binds to the collagen fibril.
2. Biglycan core-protein dissociates from the collagen fibrils and the fibril bundle decomposes into thin-fibrils.
3. Having dissociated from the fibril-bundle, the collagen type II thin-fibrils have a higher exposure of catalytic sites (increased surface area to volume ratio) and become vulnerable to collagenase and gelatinase binding, unwinding and cleavage (see above and refs. [33], [37]). Therefore, this altered collagen matrix is more fragile and readily digested by proteases.
4. We further speculate, that with the breakdown of these molecular aggregates, a host of normally hidden or low-concentration epitopes, such as PG breakdown products, collagen peptides and so on, would become present in higher concentrations than before. Theoretically, this could initiate a immunological cascade that leads to further tissue autoimmune activity and a worsening of the disease.

Lamprey notochord has a rather simple composition in comparison to mammalian cartilage, but this helps make it both an appealing and suitable model of mammalian systems for initial investigation of normal and pathological processes in cartilage at the fibrillar level. The examined interaction of anti-biglycan antibodies with the notochord ECM can serve as a model for the mechanism of rheumatoid arthritis and has potential as a simplistic RA model system. The visualization of antibody-treated tissues under different conditions inspires a speculative model of tissue destruction similar to that seen in autoimmune-induced rheumatoid arthritis, characterized by collagen matrix decomposition due to PG disruption by the anti-biglycan antibody. This proposed model highlights the crucial position of the biglycan antibody in the development of rheumatoid arthritis and our associated observations provide the first indication of what its role is beyond being a marker of this common and widespread disease.

## Materials and Methods

### Materials

Adult Sea lampreys were donated by Ludington Biological Station of the U.S. Fish and Wildlife Service and J. Ellen Marsden of the University of Vermont. Human and bovine articular cartilage, stored under physiological conditions, were provided by Tom Schmit and Vincent Wang Rush University. All samples were obtained under the appropriate institutional scientific review boards from the home institutions donating tissues.

### Initial tissue preparation

Lamprey notochord was extracted from and prepared from dissected lamprey as described previously. Bovine articular cartilage supports scraped from articular surface. Human articular cartilage plugs were harvested from donor tissues. All tissues have been stored in TBS* at 4°C at pH = 7–7.5.

### Antibody treatment for TEM and X-ray diffraction

Polyclonal biglycan antibody (Novus, epitope: DRLAIQFGNYKK) with 0.5 mg/ml concentration has been stored in 0.5 ml vials at 4°C. Thin pieces of cartilage/notochord were incubated in antibody solution for 1, 2, and 24 hours. Samples were washed in TBS 2 times for 5 min and stored in TBS pH = 7.0 at 4°C. The specificity of the antibody was previously verified by Chen et al., [39]. The solution that the tissue was incubated in the presence of antibody was collected for analysis (see SI methods S1). AFM and TEM images were recorded for intact non-antibody treated type II fibrils from lamprey notochord and bovine articular cartilage, decomposed type II fibrils from both tissues and incubating solution. Additionally microscopy data was collected from type I collagen fibrils extracted from rat tail tendon with gold conjucated antibody (and native untreated fibrils) as control data.

*Methods are continued in SI Methods S1.*

## Supporting Information

Table S1 Number of fibril/fiber species per square micron: Number of fibrils/fibers of each type: thin fibrils, thick fibers/fibrils; for each tissue: lamprey notochord, bovine cartilage and human cartilage; in each condition: native (control) and ab treated. The counts are averaged to 1 square micron of area.
(DOC)

Table S2 Size of fibril/fiber species: Average diameter of fibrils/fibers of each class for each tissue type (see methods), measured in nm. Note that the presence of large fibril-bundles inflates the determined average size for ‘thick-fibrils’.
(DOC)

Table S3 Results for sircol protocol (see SI Methods S1).
(DOC)

Table S4 Results for blyscan protocol (see SI Methods S1).
(DOC)

Methods S1 (DOC)
