# Supplementary material for: Non-Enzymatic Decomposition of Collagen Fibers by a Biglycan Antibody and a Plausible Mechanism for Rheumatoid Arthritis
Source: PLoS One. 2012 Mar 13;7(3):e32241. doi: 10.1371/journal.pone.0032241 (PMC3302792; doi:10.1371/journal.pone.0032241)
Supplement: Table S1 — Number of fibril/fiber species per square micron: Number of fibrils/fibers of each type: thin fibrils, thick fibers/fibrils; for each tissue: lamprey notochord, bovine cartilage and human cartilage; in each condition: native (control) and ab treated. The counts are averaged to 1 square micron of area. (DOC) [file pone.0032241.s001.doc]

**Table S1** Number of fibril/fiber species per square micron
