# Supplementary material for: Non-Enzymatic Decomposition of Collagen Fibers by a Biglycan Antibody and a Plausible Mechanism for Rheumatoid Arthritis
Source: PLoS One. 2012 Mar 13;7(3):e32241. doi: 10.1371/journal.pone.0032241 (PMC3302792; doi:10.1371/journal.pone.0032241)
Supplement: Table S2 — Size of fibril/fiber species: Average diameter of fibrils/fibers of each class for each tissue type (see methods), measured in nm. Note that the presence of large fibril-bundles inflates the determined average size for ‘thick-fibrils’. (DOC) [file pone.0032241.s002.doc]

**Table S2** Size of fibril / fiber species
