# Supplementary material for: Non-Enzymatic Decomposition of Collagen Fibers by a Biglycan Antibody and a Plausible Mechanism for Rheumatoid Arthritis
Source: PLoS One. 2012 Mar 13;7(3):e32241. doi: 10.1371/journal.pone.0032241 (PMC3302792; doi:10.1371/journal.pone.0032241)
Supplement: Table S3 — Results for sircol protocol (see SI Methods S1). (DOC) [file pone.0032241.s003.doc]

**Table S3** Results for sircol protocol
