# Supplementary material for: Non-Enzymatic Decomposition of Collagen Fibers by a Biglycan Antibody and a Plausible Mechanism for Rheumatoid Arthritis
Source: PLoS One. 2012 Mar 13;7(3):e32241. doi: 10.1371/journal.pone.0032241 (PMC3302792; doi:10.1371/journal.pone.0032241)
Supplement: Methods S1 — (DOC) [file pone.0032241.s005.doc]

**Supporting Information:**

MATERIALS and METHODS S1

Guanidine hydrochloride treatment

Guanidine hydrochloride was added to TBS (pH=7.0) to 4 M concentration. Tissue fragments were incubated in Guanidine hydrochloride solution for 12, 24, 48 hours. Samples were washed in TBS 2 times for 5 min and stored in TBS pH=7.0 at 4˚ C.

ABC lyase treatment

Buffer for chondroitin ABC lyase digestions - 0.1 M Tris–HCl, 0.15 M NaCl (or 50 mM NaAc), pH 7.4. Chondroitin ABC lyase digestions of cartilage tissues were performed using 0.2 units of enzyme for 1 and 12 hours at 37˚ C. Samples were washed in TBS 2 times for 5 min and stored in TBS pH=7.0 at 4˚ C.

Mechanical friction degradation

Lamprey notochord samples were mechanically fixed in stretching frame (5% stretching) at room temperature and placed in the dish with TBS. Friction was applied to topmost layer of tissue by drill with 5 mm spherical tip with 500 rpm for 30 min (when drill was off, no tension was applied to tissue). Then sample was unmounted and stored in TBS pH=7.0 at 4˚ C.

Fiber diffraction

Diffraction data was collected at BioCAT (Sector 18, Advanced Photon Sourse, Argonne National Laboratory). Data was collected in Small Angle X-ray Scattering (SAXS) mode with 2000 mm sample to detector distance, detector pixel size was 48 x 48 um (Brandeis CCD)[1]. Exposure time was 1 second for all samples.

Gold conjucation of anti-biglycan and anti-collagen antibodies for microscopy

Incubation with 30nm gold particles (Ted Pella Inc., Redding, CA) at 0.9 mg/ml in 100mM potassium bicarbonate buffer, pH 9.3 with OPSS-PEG-NHS (ortho-pyridyl disulfide-PEG-Succinimidyl Ester, Nanocs Inc), 1.5 mg/ml and 10 nm gold particle suspension.

Atomic force microscopy

Type I fibrils (rat tail tendon) were pipetted from the teased fiber ‘pull dissection’ suspension, washed in distilled water. Images were acquired with a PicoScan-3000 AFM (Molecular Imaging Co., Tempe, AZ). Type II fibrils (lamprey notochord) were pipetted from the teased dissection suspension and washed in distilled water. Imaging was carried out in fluid-tapping mode using a multimode Nanoscope IIIa AFM (Veeco Metrology), equipped with a J-scanner, using single crystal silicon tips at a resonance frequency of 300-350 kHz.

Transmission electron microscopy

Cartilage and notochord samples (native and treated with antibody, Guanidine hydrochloride, ABC lyase, and mechanical friction) have been fixed and sectioned at Electron Microscopy Center of University of Chicago (images were examined under 300 KV using an FEI Tecnai F30 microscope with a Gatan CCD digital micrograph as detector) using protocols described previously [2]. Uranyl acetate staining was used to improve contrast. TEM images of disbursed collagen fiber preparations and 30 nm gold particles conjugated to the antibody conducted by LV-EM technologists using a LV-EM5 benchtop instrument. Collagen type I and were prepared as described for AFM experiments.

Fibril count and diameter statistics

The image processing suite imagej [3] was used to measure fibril/fiber diameter and to keep track of the manual count of 'thick' and 'thin' fibrils. A thick fibril or fiber was defined as being greater than 20nm in diameter and a thin-fibril being less than this. In practice thick fibrils were significantly larger and thin fibrils significantly smaller than 20nm and had distinct and recognizable appearances. The imagej scale calibration was set against each TEM images scale bar. For lamprey notochord and bovine cartilage images, counts were made until a total (thick + fibril raw count) reach 1000 fibrils which would include several TEM micrographs. Due to the nature of the samples, far fewer images were available for human specimens, a total number of 100 fibrils was the cut off point for the manual count. The raw counts were then adjusted to reflect the area they were collected over and scaled to give a value of fibrils of each class found per square micrometer of area rounded to the nearest integer (for both count and diameter measurements).

Molecular modeling

Fibril surface conformation was based on type I collagen model reported previously [4,5], with the biglycan protein core replacing the decorin core protein (decoron) coordinates [4]. The antibody Fab fragment RCSB 2F58 (which was chosen at random from the Fab's within the RCSB database as no structure for the Fab for PG cores are currently available) was then manually docked so that it was placed adjacent to the epitope. The model is intended to be primarily illustrative, however, for completeness was energy minimized as described previously for decoron [4].

Treatment with other antibodies

TEM, AFM, and X-ray diffraction experiments were conducted with anti-Collagen Type I (Millipore, Billerica, MA) following the sample procedure as anti-biglycan treatment (above). No tissue destruction was observed. Incubation with 'normal mouse' IgG (Millipore, Billerica, MA) did not produce any detectable effect on the tissue either. Experiments were conducted for a second anti-bigylcan antibody (Abnova Walnut, CA), which also demonstrated similar biglycan removing effects.

Effect of buffer conditions on tissue stability

All buffer control incubation times were the same as for corresponding experimental conditions. However fibril decomposition was observed in notochord samples stored in PBS buffer for more than 48 hours, but not for those stored in TBS or stored in PBS for only short periods of time (2-4 hours) and then washed and stored in TBS. Studies of biglycan and decorin stability in different buffer systems revealed that biglycan is more sensitive to pH and temperature changes and it is also less stable in PBS, than in other buffers compared to decorin [6]. Therefore long exposure to PBS or interaction with the biglycan/decorin antibody can cause the conformational changes of biglycan ligated to collagen, whereas decorin appears to remains stable under the same conditions (as inferred from the fact that fibrils and fibers in rat-tail tendons appear to remain intact and unchanged from the native state as determined by TEM and X-ray diffraction). We quickly became aware of this phenomenon in our investigations, observed over several years of working with these tissues, and strongly advise other researchers to be wary of the (unwanted) effects of PBS to the stability of biglycan *in situ* of animal tissues.

PBS affect follows J.E. Scott's observation of type I degradation at pH 5.7 [7], setting the precedent that apparently weak effects have great significance for fibrilllar architecture. Recent work indicates that this is due to the electrostatic / hydrogen bonding between sLRRP and fibril [4].

Antibody treatment and tissue preparation for Sircol and Blyscan protocols and protease inhibition

Collagen and GAGs concentration measurements in LN samples after incubation in TBS and antibody solution (1:100, 1 ml, TBS) by Sircol™ and Blyscan™ kits. Lamprey notochord pieces (8 mg sample sets: 1 and 2, see SI Tables S1-S4) and 50 mg (sample set 3, see SI Tables) were incubated in 1 ml of biglycan antibody solution for 48 hours at 4ºC. Concentration of antibody (Novus) was 5 ug/ml. Control sample (native) of same mass was incubated in equal volume of TBS. After incubation notochord was placed in 1 ml (sample set 1), 4 ml (sample set 2), and 5 ml (sample set 3) ml of TBS and homogenized. Both wash solution and homogenized tissue suspension was analyzed with Sircol and Blyscan [8] kits (Biocolor) in order to determine collagen and GAGs concentrations respectively. “Halt” protease inhibitor cocktail (Thermo Scientific) was used for sample set 3 [9].

**References cited**

1. Fischetti R, Stepanov S, Rosenbaum G, Barrea R, Black E, et al. (2004) The BioCAT undulator beamline 18ID: a facility for biological non-crystalline diffraction and X-ray absorption spectroscopy at the Advanced Photon Source. J Synchrotron Radiat 11: 399–405. doi:10.1107/S0909049504016760.

2. Antipova O, Orgel JPRO (2010) In Situ D-periodic Molecular Structure of Type II. Journal of Biological Chemistry 285: 7087–7096. doi:10.1074/jbc.M109.060400.

3. Abramoff M, Magelhaes P, Ram S (2004) Image Processing with ImageJ. Biophotonics International 11: 36–42.

4. Orgel JPRO, Eid A, Antipova O, Bella J, Scott JE (2009) Decorin Core Protein (Decoron) Shape Complements Collagen Fibril Surface Structure and Mediates Its Binding. PLoS ONE 4: e7028. doi:10.1371/journal.pone.0007028.

5. Perumal S, Antipova O, Orgel JP (2008) Collagen fibril architecture, domain organization, and triple-helical conformation govern its proteolysis. Proc Natl Acad Sci U S A 105: 2824–2829.

6. Krishnan P, Hocking AM, Scholtz JM, Pace CN, Holik KK, et al. (1999) Distinct secondary structures of the leucine-rich repeat proteoglycans decorin and biglycan. Glycosylation-dependent conformational stability. J Biol Chem 274: 10945–10950.

7. Scott JE (1991) Proteoglycan: collagen interactions in connective tissues. Ultrastructural, biochemical, functional and evolutionary aspects. International journal of biological macromolecules 13: 157–161.

8. Bae JY, Han DW, Matsumura K, Wakitani S, Nawata M, et al. (2009) Nonfrozen Preservation of Articular Cartilage by Epigallocatechin-3-Gallate Reversibly Regulating Cell Cycle and NF-$ąppa$B Expression. Tissue Engineering Part A 16: 595–603.

9. Bodzon-Kulakowska A, Bierczynska-Krzysik A, Dylag T, Drabik A, Suder P, et al. (2007) Methods for samples preparation in proteomic research. Journal of Chromatography B 849: 1–31.
